# Perceived stress, stress coping strategies, and post‐traumatic‐growth among healthcare professionals during COVID‐19 pandemic

**DOI:** 10.1002/nop2.1739

**Published:** 2023-04-04

**Authors:** Roghayeh Salmani, Hasan Kazemi, Mohammad Mehrtak, Sattar Mehraban, Yalda Mousazadeh

**Affiliations:** ^1^ Department of Midwifery Khalkhal University of Medical Sciences Khalkhal Iran; ^2^ Student Research Committee Khalkhal University of Medical Sciences Khalkhal Iran; ^3^ School of Medicine and Allied Medical Sciences Ardabil University of Medical Sciences Ardabil Iran; ^4^ Research Institute of Economic Studies Al‐Zahra University Tehran Iran; ^5^ Social Determinants of Health Research Center Ardabil University of Medical Sciences Ardabil Iran; ^6^ Department of Public Health Khalkhal University of Medical Sciences Khalkhal Iran

**Keywords:** COVID‐19 pandemic, health centre, hospital, perceived stress, post‐traumatic‐growth, stress coping strategies

## Abstract

**Aim:**

The purpose of this research was to identify the perceived stress, stress coping strategies, and Post‐Traumatic‐Growth (PTG) among Iranian healthcare professionals.

**Design:**

A cross‐sectional study was applied.

**Methods:**

This study was conducted among 402 healthcare professionals in northwestern Iran. Participants completed demographic, perceived stress, stress coping strategies, and PTG questionnaires. Multiple linear regression was employed to identify the predictors of perceived stress and PTG.

**Results:**

The overall score of perceived stress was calculated 30.55 (6.18). The problem‐oriented strategy was the most common stress coping by healthcare professionals (52.66 (8.72)). Also, the total score of PTG was calculated at 45.72 (30.42). Perceived stress, stress coping strategies (except problem‐oriented), and PTG scores were significantly different between hospital and health centres participants (*p*‐value < 0.05). Previous experience in critical situations, crisis‐related course, degree, age, department, and stress coping strategies were related to the stress level. Moreover, workplace, department, work experiences, and employment status were the predictors of PTG.

## 1 INTRODUCTION

On March 11, 2020, World Health Organization (WHO) characterized the situation as a pandemic of coronavirus disease 2019 (COVID‐19) (Mosolova et al., 2020). The main feature of this disease is the easy transmissibility through human‐to‐human contact. Facing this critical emergency, governments implemented unprecedented measures to curb viral transmission (Babore et al., 2020). The rapid spread of the COVID‐19 virus has caused several challenges for health professionals, including high workload, burnout, risk of infection transmission, ethical conflicts in decision‐making for patients, and inadequate protective equipment, which have caused a heightened level of stress and other mental health symptoms (Li, Yu, et al., 2020; Sanliturk, 2021; Tian et al., 2020). As in any pandemic, front‐line nurses and medical staff experienced a great deal of anxiety and depression especially in the early stages of the disease (Shen et al., 2020; Zhang, Yang, et al., 2020; Zhou et al., 2020; Zhu et al., 2020).

In addition to workplace stresses, limitations impacting daily life, such as restrictions on public outings, have reduced chances for medical professionals to release stress, further aggravating negative mental status (Venkatesh & Edirappuli, 2020). Personality characteristics effects how individuals perceived the traumatic event. On the other hand suitable and positive coping processes can help the individual to move on from their psychological struggle (Tahara et al., 2021). So, it is important to recognize coping strategies that help healthcare professionals adjust to the highly stressful circumstances associated with COVID‐19 (Tahara et al., 2021). Many studies showed that Post‐Traumatic Growth (PTG) resulting from coping with trauma and an adaptive response to the adverse trauma (Peng et al., 2021). This growth reflected in the individual's understanding of the value of life after experiencing a traumatic event (Zhang et al., 2021). Therefore, appropriate stress coping strategies can moderate stress and provide the possibility of individual growth.

## 2 BACKGROUND

Coping is considered a stabilizing factor that helps people to adapt mentally to stressful situations. The coping process is a complex response that occurs when a person tries to eliminate perceived stress or threat from the environment (Walton, 2002). There are different methods in coping with stress, such as cognitive coping, avoidance, and emotion‐focused behaviours (Montero‐Marin et al., 2014). Stress coping strategies improved psychological symptoms, life satisfaction, and general health during the 2002 severe acute respiratory syndrome (SARS) epidemic (Main et al., 2011). During the COVID‐19 pandemic, studies have been conducted regarding stress coping strategies. A study on healthcare workers showed poor mental health among 66.6% of Japanese participants. Most of workers selected an escape‐avoidance coping strategy (Tahara et al., 2021). Religious coping and desire to serve humanity and country were common coping strategies among frontline emergency health workers in Pakistan (Munawar & Choudhry, 2021). Using of coping strategies is associated with low‐stress levels (Martínez et al., 2020; Yin et al., 2018). How people psychologically adapt to stressful situations are associated with the choice of coping strategies type (Finstad et al., 2021; Sébille et al., 2016).

Coping strategies have been considered as one of the determinants in the PTG process (Tomita et al., 2017). Individuals can reach a higher level of psychological performance after exposure to stressful conditions known as PTG, according to Tedeschi and Calhoun (Karagiorgou et al., 2018; Tedeschi & Calhoun, 1996). It is defined as “positive psychological change experienced as a result of the struggle with highly challenging life circumstances” (Zhang et al., 2021). PTG occurs as a result of having the confidence to deal with stressful events (Tedeschi & Calhoun, 2004). This self‐confidence is strengthened as a result of changing life philosophy and religious beliefs, increasing social resources, and personal resources such as management techniques (Tedeschi & Calhoun, 2004).

Nurses, paramedics, and social workers experience traumatic events indirectly and then experience growth (Rhee et al., 2013). PTG has been reported to be moderate to high among nurses, physicians, social workers, and midwives (Beck et al., 2016; Lev‐Wiesel et al., 2009; Taubman‐Ben‐Ari & Weintroub, 2008). This growth has been higher among nurses than in other groups (Beck et al., 2016; Lev‐Wiesel et al., 2009; Taubman‐Ben‐Ari & Weintroub, 2008). Two studies of health workers during the COVID‐19 pandemic showed moderate to high PTG (Feingold et al., 2022; Mo et al., 2021). It is also mentioned to apply an active coping style helps to improve the level of PTG (Mo et al., 2021). The problem‐based coping such as planning, are positive factor of PTG (Tuncay & Musabak, 2015; Stutts et al., 2015). Besides, coping may be flexible across situations, and there are specific effective strategies in certain conditions (Kato, 2012). Thus, it is essential to explore the association between coping strategies and PTG during COVID‐19 pandemic.

Studies show that the strategies to cope with stress effect on stress rate, and PTG. Therefore, studying these three phenomena together can be useful. However, the available data on stress, coping strategies, PTG, and risk factors associated with them among healthcare professionals during the COVID‐19 are still relatively limited in Iran. This study aimed to determine perceived stress, coping strategies, and PTG among healthcare professionals during the COVID‐19 pandemic. We hypothesized that the type of coping strategies (Problem‐oriented, Emotion‐oriented, and Escape) associated with perceived stress and PTG. Also, individual and occupational variables effect on perceived stress and PTG. In other words, we hypothesized that the healthcare professionals who choose rational strategies to cope with stress experience lower levels of stress. Our other assumption was that choosing the right strategy can reach people to a high level of psychological functioning or PTG. Our third hypothesis was that individual variables and working conditions affect the level of stress and PTG of a person and need to be investigated. The results obtained in our study can help develop mental health support programmes for healthcare professionals during and after the pandemic in order to mitigate actual and delayed negative psychiatric consequences.

## 3 THE STUDY

### 3.1 Design

This cross‐sectional survey was conducted from January to March 2021.

### 3.2 Aim

The present study has three aims. The first aim was to determine the scores of perceived stress, the coping strategies, and PTG among healthcare professionals during COVID‐19 pandemic. The second aim was to determine individual and occupational variables effect on perceived stress and PTG. The third aim was to determine the type of coping strategies effect on perceived stress and PTG.

### 3.3 Sampling and data collection

A survey was conducted in a public hospital and ten health centres in Ardabil province, northwestern Iran, which were selected as referral centres for patients with COVID‐19. The study population included 402 healthcare professionals working the morning, evening, and night shifts, with participants selected based on census sampling. In total, 450 questionnaires were circulated by researchers from the 25th of January to 15th of March, 2021. Participants were asked to complete and return the questionnaires within 20 days.

### 3.4 Survey tools

The first questionnaire was about demographic information comprising of age, sex, education level, marital status, type of employment, hospital and department name, city, work experiences (in years), shift type, having children, passing a crisis‐related course, continuous presence during the COVID‐19 outbreak, direct exposure to the patients with COVID‐19, and previous experience of dealing with critical situations.

The second questionnaire was developed by Cohen et al. (1983), named the perceived stress scale (Cohen et al., 1983), which consists of 14 items. Scores are given on a five‐point Likert scale. The lowest and highest achievable scores are 0 and 56, respectively, and a higher score indicates more stress. Cohen et al. confirmed the reliability of this questionnaire by Cronbach's alpha coefficient of 0.75 (Cohen et al., 1983). In addition, Maroufizadeh et al. in Iran, confirmed that the 14‐item perceived stress scale has satisfactory psychometric properties for measuring stress. Cronbach's alpha coefficient in this study was 0.90 (Maroufizadeh et al., 2014).

The third questionnaire used in this study was the Coping with Stressful Situations questionnaire by Endler and Parker, consisting of 48 items (Endler & Parker, 1990a). The questionnaire covers three main areas of coping behaviours (each area with 16 items) including Problem‐oriented confrontation, Emotion‐oriented confrontation, and Confrontation avoidance or escape from the problem. The items of this questionnaire are scored based on a 5‐point Likert scale. The score of each of the three confrontational behaviours, that is, problem‐oriented, Emotion‐oriented, and Confrontation avoidance or escape from the problem. The total internal consistency coefficient is reported 0.92 in the main sample of the Endler and Parker (1990b). The Cronbach's alpha coefficient of the Persian translation of this questionnaire was calculated at 0.83 (Ghoreishi Rad, 2010).

Another tool used in this study was the Post‐Traumatic Growth Inventory (PTGI) by Tedeschi and Calhoun (1996) consisting of 21 items in the five domains of growth: (1) relating to others better (seven items); (2) recognizing new possibilities (five items); (3) a greater sense of personal strength (four items); (4) spiritual change (two items); and (5) greater appreciation of life (three items). The items are scored based on a 5‐point Likert scale. The subject scores range from 0 to 105. There is excellent internal and test–retest reliability among Western and Asian samples (Jin et al., 2014; Tedeschi & Calhoun, 1996). In the study of Najafi Gharehasani et al., the total alpha coefficient was 0.93 among Iranian nurses (Najafi Gharehasani et al., 2020).

### 3.5 Analysis

To analyse the data, Statistical Package for Social Science (SPSS) software, version 25 (IBM Corp.) was used. The respondents' demographic characteristics were presented using descriptive statistics: frequency/percentages, mean, median, and standard deviation (SD). Also, the scores of questionnaires have been calculated according to each dimension and then analysed in the software using descriptive data analysis by means, percentage, and standard deviation. Normality was checked via the Shapiro–Wilk W test. Multiple linear regression was employed to identify the predictors of perceived stress and PTG.

### 3.6 Ethical considerations

Ethical permission was obtained from the Ethics Committee of Ardabil University of Medical Science (ethical code: IR.ARUMS.REC.1399.430). Informed consent: All participants were informed about the aim of the study. Written informed consent was obtained from the participants who could withdraw from the study at any stage.

## 4 RESULTS

### 4.1 Participants

From 450 distributed questionnaires, about 402 responses were received during three months. The overall response rate was 89.33%.

The majority of subjects were female (74.1%) and married (75.6%). The participants with children were 60.95%. The majority of the samples held a Bachelor's degree (75.12%) and had fixed shifts (52.5%). The mean age of the participants was 35.1 (*SD* = 7.7) years, and the average experience was 10.5 (*SD* = 7.9) years. most subjects had full‐time jobs in the hospital (47.76%) in the hospital (50.75%). Characteristics of the samples are summarized in Table 1.

**TABLE 1:** General characteristics of participants (*N* = 402).

Variables	*N* (%)
Gender	
Female	298 (74.1)
Male	104 (25.8)
Marital status	
Single	98 (24.4)
Married	304 (75.6)
Employment status	
Official	192 (47.76)
Semi‐official	35 (8.71)
Contractual	89 (22.14)
Corporative	37 (9.2)
Temporary (2 or 4 years)	49 (12.19)
Education level	
Diploma	52 (12.94)
Bachelor's	302 (75.12)
Master's	26 (6.47)
Professional Doctorate	22 (5.47)
Workplace	
Hospital	204 (50.75)
Health Centre	198 (49.25)
Department	
Inpatient	99 (24.63)
Clinic	17 (4.23)
Emergency	28 (6.97)
Pre‐hospital	14 (3.48)
Surgery Room	9 (4.73)
Para clinic^a^	36 (8.96)
Health Centre^b^	189 (47.01)
Shift	
Rotational	191 (47.5)
Fixed	211 (52.5)
Having children	
Yes	245 (60.95)
No	157 (39.05)
City	
Ardabil	42 (10.45)
Bilehsavar	45 (11.19)
Khalkhal	238 (59.2)
Meshkinshahr	38 (9.45)
Parsabad	39 (9.7)
Age (in years)	
20–30	143 (35.6)
31–40	163 (40.5)
41–50	86 (21.4)
>50	10 (2.5)
Work experience (in years)	
<5	160 (39.8)
6–10	65 (16.17)
11–15	78 (19.4)
16–20	44 (10.95)
21–25	30 (7.46)
26–30	25 (6.22)
Crisis‐related course	
Yes	291 (44.9)
No	111 (55.1)
Continuous presence in workplace during the COVID‐19 outbreak	
Yes	388 (96.52)
No	14 (3.48)
Direct exposure to the patients with COVID‐19	
Yes	340 (84.58)
No	62 (15.42)
Having critical situations (formerly)	
Yes	248 (70.65)
No	118 (29.35)

^a^ Para‐clinical department included radiology, laboratory, and pharmacy.

^b^ Different sections of health centres were studied in present research.

### 4.2 Perceived stress, stress coping strategies, and PTG scores

The overall score of the perceived stress was 30.55 ± 6.18. According to the results, the most common stress coping strategy by healthcare professionals was the problem‐oriented (52.66 ± 8.72). Also, the total score of PTG was calculated (45.72 ± 30.42). Relating to others had the highest score. The scores of the studied variables are summarized in Table 2. A statistically significant difference was found in the scores of perceived stress, stress coping strategies (except problem‐oriented) between hospital and health centres participants.

**TABLE 2:** The scores of perceived stress, stress coping strategies, and PTG.

Variable	Hospital				Health centre				Mean* difference	*p*‐value
	Mean (SD)	Median	Minimum	Maximum	Mean (SD)	Median	Minimum	Maximum		
Perceived stress	31.25 (6.22)	31	9	56	29.82 (6.08)	30	1	52	1.42**	0.02
Stress coping strategies										
Problem‐oriented	52.44 (8.51)	51	34	76	52.89 (8.96)	54	29	76	−4.48	0.60
Emotional‐oriented	42.42 (8.94)	42	24	71	39.51 (9.46)	39	16	72	2.90***	0.002
Avoidance	45.47 (7.96)	45	24	71	43.58 (7.99)	43	24	67	1.89**	0.01
PTG										
	33.76 (29.92)	20.5	0	104	58.03 (25.71)	62.5	0	105	−24.26***	0.000
Recognizing new possibilities	8.25 (7.61)	6	0	25	14.19 (6.55)	15	0	25		
Relating to others	10.75 (9.43)	7	0	34	18.08 (8.61)	20	0	35		
Appreciation of life	5.03 (4.69)	3	0	15	8.8 (4.23)	10	0	15		
Personal strength	6.48 (6.06)	4	0	20	11.51 (5.38)	12	0	20		
Spiritual change	3.25 (3.34)	2	0	10	5.45 (3.08)	6	0	10		

* T‐test was applied

** *p*‐value < 0.05

*** *p*‐value < 0.01.

Table 3 shows that 294 of the participants adopted problem‐oriented approach. The mean (*SD*) perceived stress and PTG were 27.7 ± 8.03 and 46 ± 31, respectively.

**TABLE 3:** The scores of the perceived stress and PTG according to stress coping strategies.

Variable	Frequency (%)	Mean (SD)^a^	Mean (SD)^b^
Stress coping strategies			
Problem‐oriented	294 (73.13)	27.7 (8.03)	46 (31)
Emotional‐oriented	42 (10.45)	31.31 (8.05)	44 (28)
Avoidance	43 (10.7)	30.63 (5.63)	42 (28)
Unknown	23 (5.73)		

^a^ Perceived stress

^b^ PTG.

### 4.3 Regression model

Multiple linear regression models were employed to examine the effect of each individual and occupational variable as well as coping strategies on perceived stress and PTG. Based on the results of the regression model, the type of adopted approach in the face of stress on perceptual stress was significant. By changing the approach from avoidance to emotion‐oriented and problem‐oriented, the amount of perceptual stress has increased by 4.07 and 3.21 units, respectively.

Table 4 shows a significant relationship between previous critical situations and perceptual stress so that the level of perceptual stress increased by 1.5 units. In addition, people with a crisis‐related course expressed more 1.5 units on average on the perceptual stress. Participants with a bachelor's and professional doctorate had more perceptual stress than the reference group (diploma). By changing the status of individuals from the diploma group to the bachelor group and professional doctorate, their perceptual stress levels increased by 2.17 and 4.06 units, respectively. Healthcare professionals demonstrated 2.33 units of more stress in para‐clinical departments compared to the inpatient wards. Finally, the results show that with the increase of each unit in the age of the participants, the level of perceptual stress decreases −0.16 units. No significant relationship was found between the other variables.

**TABLE 4:** Regression model of individual and occupational variables and perceived stress.

Variable	Regression coefficient (β)	Standard deviation	T statistics	*p*‐value	Confidence interval	
					Lower	Upper
Copping strategies						
Problem‐oriented	4.07	2.03	2*	0.04	0.06	8.07
Emotional‐oriented	3.21	1.74	1.84**	0.06	−0.22	6.65
Unknown	2.86	3.06	0.94	0.35	−3.15	8.88
Avoidance	Reference	Reference	Reference	Reference	Reference	Reference
Workplace						
Hospital	1.46	1.55	0.94	0.34	−1.59	4.53
Health centre	Reference	Reference	Reference	Reference	Reference	Reference
Department						
Clinic	−1.23	1.53	−0.8	0.42	−4.24	1.77
Emergency	−0.63	0.96	−0.65	0.51	−2.53	1.27
Pre‐hospital	1.27	1.75	0.72	0.46	−2.17	4.71
Health centre	0.52	1.67	0.31	0.75	−2.76	3.81
Surgery room	−1.36	1.33	−1.02	0.30	−3.99	1.26
Para clinic	2.43	1.42	1.71**	0.08	−0.36	5.22
Inpatient	Reference	Reference	Reference	Reference	Reference	Reference
Having children						
Yes	1.19	1.03	1.15	0.24	−0.84	3.22
No	Reference	Reference	Reference	Reference	Reference	Reference
Shift						
Rotational	−0.35	1.08	−0.33	0.74	−2.48	1.77
Fixed	Reference	Reference	Reference	Reference	Reference	Reference
Marital status						
Single	−0.31	1.1	−0.29	0.77	−2.48	1.84
Married	Reference	Reference	Reference	Reference	Reference	Reference
Sex						
Male	0.45	0.92	0.49	0.62	−1.36	2.27
Female	Reference	Reference	Reference	Reference	Reference	Reference
Work experience (in years)						
6–10	−3.16	2.56	−1.24	0.21	−8.2	1.87
11–15	−1.9	2.2	−0.86	0.39	−6.24	2.43
16–20	−2.03	2.03	−1	0.31	−6.04	1.97
21–25	−2.64	1.83	−1.44	0.15	−6.24	0.95
26–30	−1.19	2.2	−0.54	0.58	−5.52	3.13
<5	Reference	Reference	Reference	Reference	Reference	Reference
Having critical situations (formerly)						
Yes	1.5	0.64	2.35*	0.02	0.24	2.76
No	Reference	Reference	Reference	Reference	Reference	Reference
Crisis‐related course						
Yes	1.5	0.73	2.04*	0.04	0.05	2.95
No	Reference	Reference	Reference	Reference	Reference	Reference
Direct exposure to the patients with COVID‐19						
Yes	−0.21	0.84	−0.25	0.79	−1.86	1.44
No	Reference	Reference	Reference	Reference	Reference	Reference
Continuous presence in workplace during the COVID‐19 outbreak						
Yes	0.71	1.71	0.42	0.67	−2.65	4.09
No	Reference	Reference	Reference	Reference	Reference	Reference
Education level						
Bachelor's	2.17	1.18	1.84**	0.06	−0.15	4.50
Master's	0.87	1.51	0.57	0.56	−2.11	3.86
Professional Doctorate	4.06	1.93	2.1*	0.03	0.25	7.86
Diploma	Reference	Reference	Reference	Reference	Reference	Reference
Employment status						
Corporative	−0.21	1.09	−0.2	0.84	−2.36	1.93
Contractual	−0.48	0.96	−0.5	0.61	−2.38	1.42
Semi‐official	1.2	1.38	0.87	0.38	−1.51	3.92
Official	1.4	1.49	0.94	0.34	−1.52	4.34
Temporary	Reference	Reference	Reference	Reference	Reference	Reference
Age	−0.16	0.09	−1.76**	0.08	−0.34	0.01

*Note*: R squared = 0.11.

* Significant at the 0.05 level

** Significant at the 0.1 level.

Table 5 shows the relationship between stress coping strategies and PTG was not significant. The results show that with the change in the status of the personnel department from the inpatient department to the emergency department, the PTG rate increased by 20 units. Unlike the emergency ward, the samples of the pre‐hospital and surgery room expressed lower PTG (−18.42 and −13.71 units, respectively) compared to the inpatient wards. The results show that people who worked in the hospital experienced significantly less growth than the people who worked in health centres (−21.17 units, *p*‐value = 0.003). Participants with a work background of 6–10 years experienced less growth than people with a work experience of fewer than five years (−25.22 units, *p*‐value = 0.02). By changing the status of employment from the temporary group to the contractual group, PTG increased by an average of 10.59 units. No significant relationship was found between the other variables.

**TABLE 5:** Regression model of individual and occupational variables and PTG.

Variable	Regression coefficient (β)	Standard deviation	T statistics	*p*‐value	Confidence interval	
					Lower	Upper
Copping strategies						
Problem‐oriented	2.38	7.42	0.32	0.749	−12.22	16.98
Emotional‐oriented	5.04	6.4	0.79	0.431	−7.55	17.64
Unknown	−7.89	10.2	−0.77	0.44	−27.95	12.17
Avoidance	Reference	Reference	Reference	Reference	Reference	Reference
Workplace						
Hospital	−21.17	7.11	−2.988***	0.003	−35.16	−7.18
Health centre	Reference	Reference	Reference	Reference	Reference	Reference
Department						
Clinic	−6.59	7.41	−0.89	0.37	−21.17	7.98
Emergency	20.79	5.87	3.54***	0	9.23	32.34
Pre‐hospital	−18.42	8.38	−2.2*	0.02	−34.91	−1.93
Health centre	−1.99	7.79	−0.26	0.79	−17.31	13.32
Surgery room	−13.71	6.83	−2.01*	0.04	−27.15	−0.27
Para clinic	−8.02	5.72	−1.4	0.16	−19.28	3.23
Inpatient	Reference	Reference	Reference	Reference	Reference	Reference
Having children						
Yes	−2.95	4.93	−0.6	0.55	−12.64	6.74
No	Reference	Reference	Reference	Reference	Reference	Reference
Shift						
Rotational	−0.71	3.97	−0.18	0.857	−8.53	7.1
Fixed	Reference	Reference	Reference	Reference	Reference	Reference
Marital status						
Single	4.99	4.56	1.09	0.27	−3.98	13.97
Married	Reference	Reference	Reference	Reference	Reference	Reference
Sex						
Male	−5.55	3.55	−1.56	0.12	−12.55	1.44
Female	Reference	Reference	Reference	Reference	Reference	Reference
Work experience (in years)						
6–10	−25.22	11.36	−2.22*	0.02	−47.56	−2.88
11–15	−12.32	9.49	−1.3	0.19	−30.98	6.33
16–20	−11.78	8.44	−1.4	0.16	−28.37	4.81
21–25	−9.36	7.79	−1.2	0.23	−24.68	5.96
26–30	0.18	7.53	0.02	0.98	−14.64	15
<5	Reference	Reference	Reference	Reference	Reference	Reference
Having critical situations (formerly)						
Yes	−3.31	3.53	−0.94	0.34	−10.26	3.62
No	Reference	Reference	Reference	Reference	Reference	Reference
Crisis‐related course						
Yes	0.75	3.68	0.21	0.83	−6.48	8
No	Reference	Reference	Reference	Reference	Reference	Reference
Direct exposure to the patients with COVID‐19						
Yes	−5.46	4.23	−1.29	0.19	−13.8	2.86
No	Reference	Reference	Reference	Reference	Reference	Reference
Continuous presence in workplace during the COVID‐19 outbreak						
Yes	−2.34	7.72	−0.3	0.76	−17.53	12.84
No	Reference	Reference	Reference	Reference	Reference	Reference
Education level						
Bachelor's	3.86	5.02	0.77	0.44	−6.01	13.74
Master's	8.7	7.37	1.18	0.23	−5.79	23.19
Professional Doctorate	5.34	7.79	0.69	0.49	−9.98	20.67
Diploma	Reference	Reference	Reference	Reference	Reference	Reference
Employment status						
Corporative	5.42	5.65	0.96	0.33	−5.68	1.93
Contractual	10.59	4.51	2.35*	0.01	1.72	1.42
Semi‐official	5.58	6.38	0.88	0.38	−6.96	3.92
Official	8.85	6.25	1.42	0.15	−3.44	4.34
Temporary	Reference	Reference	Reference	Reference	Reference	Reference
Age	0.2	0.44	0.45	0.65	−0.67	1.07

*Note*: R squared = 0.3.

* Significant at the 0.05 level

*** Significant at the 0.01.

## 5 DISCUSSION

This study was conducted on exploring the perceived stress, stress coping strategies, and PTG among Iranian healthcare professionals. There were higher than average of perceived stress among the participants. About 80% of the participants took a problem‐oriented approach to coping with stress. In addition, the total score of PTG was calculated as low to moderate. Given the pandemic status of COVID‐19 disease, which affects all economic, political, and social aspects of the world, there are adverse psychological impacts of this viral disease on the mental health of people (Li, Wang, et al., 2020).

In this study, the healthcare professionals, especially in the hospital section, have experienced much stress. Other similar studies have reported moderate to high stress level among frontline personnel following the COVID‐19 virus pandemic (Babore et al., 2020; Huang et al., 2020; Sanliturk, 2021; Zhang, Wang, et al., 2020). Cheong et al. and Zhong et al. reported that about a third of those with SARS were the health staff in two separate studies, who had stress and anxiety (Cheong & Lee, 2004; Zhong et al., 2003). Sirati et al. concluded in their study that more than 90% of the subjects experienced moderate to severe stress, more severely in the health staff than the non‐health staff (Sirati Nir et al., 2020). Other studies in Iran also showed that the level of anxiety and depression of healthcare workers (physicians and nurses) was higher than the general level of society during the COVID‐19 pandemic (Hassannia et al., 2020; Kaveh et al., 2020). In the present study, considering that the hospital section staff has direct contact with patients of more critical conditions, they have experienced more stress.

Previous experience of critical situations, crisis‐related training courses, degree, department, and age were closely related to the level of stress perceived in this study. In the study of Sanlıtürk's, the level of occupational stress was affected by gender, the number of children, years of experience in intensive care, and the type of work shift (Sanliturk, 2021). Sirati et al. showed that men and people taking certain medications experience more stress (Sirati Nir et al., 2020). In some studies, younger people, such as students, were more likely to be exposed to generalized anxiety disorder during the development of the COVID‐19 virus (Huang & Zhao, 2020; Qiu et al., 2020). These findings are consistent with the results of the present study, which indicate that stress decreases with age. It might be that older people can more efficiently manage stressful situations. In this study, previous experience of critical situations and crisis‐related courses led to higher levels of stress indicating impracticality of such courses. On the other hand, being aware of a critical situation or higher education may have led to more stress. Another reason for the difference in results with previous studies may be due to differences in working conditions and studied job and work variables.

According to the results, problem‐oriented approach was the most common coping strategy. However, in two studies on nurses and nursing students, it has been demonstrated that avoidance or secrecy coping mechanisms were the dominant coping strategies against their physio‐psycho‐social status (Matos et al., 2010; Wang et al., 2015). Rolin et al. showed that healthcare workers endorsing problem‐focused coping strategies had lower levels of the perceived threat and higher levels of perceived control in their response to the COVID‐19 pandemic (Rolin et al., 2021). In the present study, a statistically significant relationship was found between coping strategies and stress levels. Similarly, Hasan et al. concluded a significant relationship between the perceived stress level and the adoption of a coping mechanism (Hasan et al., 2018). In another study, Morse et al. found that the coping strategy could help individuals face and handle a stressful situation, which can affect the level of general health (Morse et al., 2012). Unexpectedly, even though the study participants employed effective coping strategies (problem‐oriented), they experienced higher levels of stress, which might be related to the unknown nature of the disease. On the other hand, having insufficient knowledge about a phenomenon can heighten the stress level.

In this study, PTG was low to moderate among participants with its rate higher in health centres than in hospitals. Pan Cui et al. reported that the PTG of frontline nurses was at a medium to high level and influenced by working years, self‐confidence in frontline work, awareness of risk, psychological intervention or training and deliberate rumination (Pan Cui et al., 2021). Peng et al. also found that the average score of PTGI in frontline nurses was moderate. The PTG score increased significantly in individuals who had support from family members and friends, had psychological comfort and had children (Peng et al., 2021). In this study, PTG was related to department, workplace, work experience, and employment status. However, no statistical correlation was found between PTG and the coping strategies. It seems that the personnel with more stable employment status have paid more attention to the issue of suitable stress management. Unlike the present study, positive coping strategies were significantly associated with elevated PTG in several studies (Finstad et al., 2021; Kalaitzaki et al., 2021; Rajandram et al., 2011).

The findings of the present study show the mental state of a large number of healthcare professionals. This information might be used to design and implement programmes to reduce occupational stress, strengthen morale and develop rational decision‐making strategies in critical situations. Also, mental health experts might provide counselling services for improving mental state and positive attitude style. Two earlier studies showed that teaching job coping strategies can reduce job stress (Ebert et al., 2016; Sohrabi et al., 2019). The results of the study of Khatoni et al. showed that stress immunization training on the level of job stress of nurses working in selected military hospitals was not associated with a significant reduction in their job stress (Khatoni et al., 2020). Xu et al. reported that the scores of PTG and its subscales were significantly higher after intervention (Xu et al., 2016). Further studies can focus on the accuracy of the training practicality.

### 5.1 Strengths and limitations

There are several strengths in this study. First, the issues of perceived stress, coping strategies, and PTG are the three fundamental problems, rarely studied together in studies conducted in Iran. In this study, the studied healthcare professionals were among the vulnerable occupations, especially during the COVID‐19 pandemic, which might exacerbate the rate of stress. Second, the participants were satisfied with the content of the studied factors in this study. On the other hand, further studies may be required in other settings and among other healthcare staff to generalize the findings. Furthermore, other longitudinal studies are required for a more accurate assessment of the constructs covered in this study.

## 6 CONCLUSION

Considering that the stress in health professional leads to a quantitative and qualitative drop in health services, identifying the stress level in healthcare professionals in the work environment encourages strategies to reduce the effects of stress and improve the quality of service delivery. It is clear from the results of this study that the Iranian healthcare professionals experienced more rate of stress due to the nature of their job and the unknown nature of the new pandemic. It highlights the importance of supportive programmes. Nursing managers can reduce stress as much as possible by providing appropriate protective equipment, division of labour, and working hours. However, many participants used a problem‐based strategy to deal with stress, indicating a rational approach to the issue. In addition, many people studied especially in health centres expressed a higher level of mental function in this study (high PTG). Social support and professional psychological intervention should be applied to further improve the PTG level. The leaders should pay attention to PTG and the factors influencing it and offer solutions to retain mental health. In this way, they can reduce tensions in the work environment. Given that many participants have high levels of stress despite previous experience, related training courses and positive coping strategies, it seems that designing practical training courses, specifying relevant concepts in syllabuses, support strategies, and appropriate counselling ameliorate the performance of the healthcare professionals.

## FUNDING INFORMATION

The current study was financially supported by Ardabil University of Medical Sciences.

## CONFLICT OF INTEREST STATEMENT

The authors declare that there is no conflict of interest.

## Data Availability

The datasets generated and/or analysed during the current study are available from the corresponding author on reasonable request.
